# Concept of Sustained Ordering and an ATP-related Mechanism of Life’s Origin

**DOI:** 10.3390/ijms10052019

**Published:** 2009-05-06

**Authors:** Erik M. Galimov

**Affiliations:** V.I. Vernadsky Institute of Geochemistry and Analytical Chemistry RAS 119991, Moscow, Kosygin str., 19, GEOKHI, Russia; E-Mail: galimov@geohki.ru

**Keywords:** Origin of Life, Concept of Ordering, ATP

## Abstract

This paper shows that the steady state of a system of conjugated reactions, which are characterized by disproportionation of entropy and proceed in the domain of linear interactions, is an attractor of ordering. Such systems are primed to produce ordering, and life is a specific manifestation of the sustained ordering inherent to the chemistry of carbon. The adenosine triphospate (ATP) molecule has properties which makes ATP hydrolysis to be most appropriate to form such a system in primitive world. Hence, ATP is suggested to play a key role in prebiological evolution. Principles of the origin and evolution of life following from the concept of ordering are stated.

## 1. Introduction

The most evident and general characteristic of life is its highly ordered state and increase of order, both during the life of an individual organism and on the scale of the entire history of the evolution of life.

Currently, Darwinism is a main evolutionary concept. As a theory of adaptation of organisms to variable environments, Darwinism is confirmed by numerous examples. However, it should be emphasized that natural selection is not an agent of ordering. Natural selection does not create anything new. Selection preserves that which already exists, that which is most resistant to processes of disordering. The ordering itself in Darwinism is regarded as an accidental favorable change (positive mutation in modern terms) fixed by natural selection. At the level of simple molecules, which played a role at the pre-biological stage, “natural selection” leads to equilibrium. Therefore it does not help us to understand the origin of life. Dawkins [1] believes that the problem of the origin of life can be approached by assuming a low-probability case, when a structure (in fact, the simplest organism) capable of further development by natural selection emerges spontaneously. Similarly, Arrhenius [2, p. 204] argues that life begins from random interaction and growth of macromolecules. When they “reach the large size that eventually permit biofunctionality, then the system emerges from chaos into a Darwinian selection regime, governed by rules other than those of chance”. Thus the apologists of Darwinism assume that Darwinian selection cannot explain the origin of life, and believe that life emerged and developed to the certain level by chance. Obviously the difficulty stems from the fact that Darwinism is not a theory of ordering.

Numerous models of self-organization have been suggested [3–8]. However, all these models include natural selection as a driving force. It has been shown in non-equilibrium thermodynamics that in irreversible processes far away from equilibrium spatially correlated forms (dissipative structures) emerge, and evolution can proceed through consecutive instabilities by bifurcations [9]. Prigogine suggested that such a mechanism is inherent to the origin and evolution of life [10]. Although this physical phenomenon exists and some features of evolution may be described on this theoretical basis, it is unlikely to be applicable to biological ordering in general. Eigen noted that: “macroscopic ordering in geometric space (which implies the formation of dissipative structures) has few analogies with functional ordering occurring in biological objects” [3]. Eigen’s own concept of evolution, related to notion of value of information, also met criticism: “there is no value without goal: the question about value of information emerges only when the goal is determined” [6, p. 11]. But evolution has no goal.

The extreme alternative to Darwinism is the concept of a Designer (which suggests existence of a goal!). Anti-Darwinist Behe [11] analyzed the biological systems of “irreducible complexity” (which make sense only taking as a whole) and concluded that such systems could not be formed by Darwinian evolution, Noble Laureate C. De Duve [12] wrote in his book: “Why not imagine a God who, from the start, created a world capable of giving rise to life by the sole unfolding of natural laws of His own devising?” However it is not sufficient to have a design. The design must be implemented. If one assumes that there has been a creator behind each system of “irreducible complexity”, each new species, and each individual, then the creator must have a set of instruments, which allow it to direct every molecule at each moment in time and at each point in space. Such an idea of a creator coincides with our understanding of Nature, where the “instruments” are the natural laws.

Eventually, the problem is reduced to the question: Is there a mechanism of natural ordering, which explains emergence and evolution of life? This paper presents an approach, different of those previously suggested, and describes a model of ordering which is relevant to the phenomenon of life in Nature.

## 2. The concept of ordering

### 2.1. Definition of ordering

According to the statistical-mechanics interpretation of entropy, the entropy *S* is proportional to the logarithm of the number W of states that the system can acquire:

S=klnW

The proportionality coefficient is the Boltzmann constant *k*. In thermodynamics entropy has the dimension of thermal unit to temperature degree. However the notion of entropy goes beyond the strict scope of thermodynamics, *e.g.* it is used in information theory where it carries the name of Gibbs-Shannon entropy. The coefficient *k* is omitted in this case, and entropy has a non-dimensional value.

In any interpretation ordering is reduced to restriction of freedom. Each constraint applied to a system increases its ordering. Thus ordering is restriction of freedom: freedom of movement, freedom of interaction, and production of forms, which are able to restrict freedom.

### 2.2. Disproportionation of entropy

It is well known that entropy may decrease in an open system. However there should be a mechanism by which this possibility would be put in force. Note that decrease of entropy may occur in isolated system as well.

All non-equilibrium processes are characterized by production of entropy:

$$\frac{\partial S}{\partial t}=\sum X_{k}J_{k}>0$$

where X_k_ and J_k_ represent generalized forces and fluxes respectively. In chemistry, flux is determined by change in concentration of the components (*i.e.* the reaction rate). Force is an agent that depends on the misbalance of chemical potentials of the components. Concentration always changes so as to reduce the misbalance, which results in equilibrium. Therefore, the force and the flux have the same signs, and their product is positive.

However, if the process involves two conjugated processes X_1_J_1_ and X_2_J_2_, then one of them can take place with negative sign, provided that the other process produces more entropy than is required to compensate this decrease. Then *disproportionation of entropy* occurs. At that the total entropy production remains positive:

$$\begin{array}{l} {\text{X}}_{1}{\text{J}}_{1}+(-{\text{X}}_{2}{\text{J}}_{2})>0\\ \Delta \text{S}\;=\;\Delta {\text{S}}_{1}-\Delta {\text{S}}_{2}>0 \end{array}$$

The negative sign denotes ordering. It should be stressed that a *microscopic conjugation is an indispensable condition of the entropy disproportionation*. The conjugated processes are actually just different sides of the same process. Thus, the disordering process makes the coupled ordering process possible. The episodes of ordering conjugated with disordering are omnipresent in Nature. However, this does not yet account for the sustained ordering displayed by life.

### 2.3. Attractor

Attractor is defined as a final stable state, which a process is going to. When a system is brought out of a stable state, an attractor strives to bring it back to the initial state. Equilibrium state is the attractor of the disordering process. According to thermodynamics all processes tend to reach the minimal free energy and the maximal entropy. This is an equilibrium state. If a system is taken out of this state, it tends to return to it.

Is there an attractor for the ordering process?

I argue that such an attractor appears in a *steady state of conjugated processes, which involve entropy disproportionation and proceeds in the domain of linear interactions.*

### 2.4. Steady state

A steady state is a very special state. It has some properties of an equilibrium, but it is fundamentally different from an equilibrium state. In a steady state concentrations of reagents and products remain constant, like in the case of equilibrium, but – for different causes. In a steady state the reagents are continuously supplied into the system, while the products are taken away from the system at the same rate. As a result, concentrations of the components of the system remain unchanged.

As the concentrations do not change, the entropy remains constant:

ΔS=0

On the other hand, maintenance of the steady state requires continuous consumption of energy. Therefore, as in any non-equilibrium system, the production of entropy in a steady state is positive:

∂Si/∂t>0

In order to satisfy the condition Δ*S*=0, the positive production of entropy must be balanced by a flux of the negative entropy from environment [11]:

δSi/∂t=∂Se/∂t

### 2.5. Production of ordering in a steady state of conjugated reactions with disproportionation of entropy

In chemistry conjugation occurs when a reactant of one of the reactions acts as a product in the other one:

$$\begin{array}{c} \text{A}+\text{I}\to \text{B}+\text{P};\\ \text{M}+\text{N}\to \text{I}+\text{MN}, \end{array}$$

Where I here is a conjugating component.

If the system is presented by conjugated reactions then:

$${\left(\frac{\partial Si}{\partial t}\right)}_{1}+{\left(-\frac{\partial Si}{\partial t}\right)}_{2}=\frac{\partial Se}{\partial t}$$

where the symbol (1) is used for an energy-providing and entropy-producing reaction, and symbol (2) – for the negative entropy producing (ordering) reaction.

It is an essential feature of the system in a steady state that in this state its entropy production is minimized. This theorem was proven by Prigogine [12]. In contrast to an equilibrium state, when the entropy does not change reaching a maximum, in a steady state the entropy increases but the rate of the increase is minimal.

Hence, in a steady state:

$${\left[{\left(\frac{\partial Si}{\partial t}\right)}_{1}+{\left(-\frac{\partial Si}{\partial t}\right)}_{2}\right]}_{\text{min}}=\frac{\partial Se}{\partial t}$$

Moreover, when the system is taken out of this state, it strives to return to it. In other words, the state with minimal entropy production is an *attractor*. If a system deviates from the steady state its production of entropy increases:

$${\left(\frac{\partial Si}{\partial t}\right)}_{1}^{/}+{\left(-\frac{\partial Si}{\partial t}\right)}_{2}^{/}>\frac{\partial Se^{\prime }}{\partial t}$$

Any components of entropy production may change (symbol′). However, if the energy supplying reaction (1) is fixed, that is:

$${\left(\frac{\partial Si}{\partial t}\right)}_{1}^{\prime }={\left(\frac{\partial Si}{\partial t}\right)}_{1}$$

then the only way to return to the steady state is decrease of ${\left(-\frac{\partial Si}{\partial t}\right)}_{2}^{/}$ value. Hence, in force of the *principle of minimal production of entropy in* a steady state the system makes the next step of ordering:

$${\left[{\left(-\frac{\partial Si}{\partial t}\right)}_{2}^{/}\right]}_{\text{min}}<{\left(-\frac{\partial Si}{\partial t}\right)}_{2}^{/}$$

Since the attractor does exist the ordering becomes obligatory. Moreover, to get minimum production of entropy the system seeks a way to maximize ordering. The theorem on the minimal entropy is valid only in the linear interactions range. The system should not be brought too far away from the steady state to avoid a non-linear response.

It is easy to note that living organisms have the properties of such systems. They exist close to steady state; need a continuous energy input, feature matter exchange (that is metabolism), and die when taken too far off the steady state.

The distinctive feature of the suggested model is the proof that a system isolated with its environment under certain condition (stated above) obligatorily produces ordering. This is most relevant to emerging and evolution of life in Nature. I argue that ordering can be treated in terms of linear thermodynamics. The isotope distribution pattern in biological systems gives evidence of the linear character of the enzyme-controlled biochemical processes [13,14]. We will discuss the case of evolution later.

## 3. Significance of ATP

**Figure 1.**
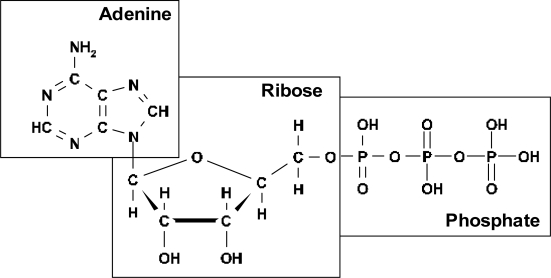
Adenosine triphospate.

I assume that the best candidate for realization of this mechanism of ordering in pre-biological times was adenosine triphosphate (ATP) (Figure 1).

There are several reasons for distinguishing the pre-biological role of ATP. ATP hydrolysis is accompanied by a considerable release of free energy, about Δ*G* = −31 kJ/mol. The participation of ATP can therefore supply energy to chemical reactions that cannot occur spontaneously. In modern organisms, ATP participates in all energy-absorbing biochemical reactions. It accumulates various types of external energy in the process of phosphorylation and transforms them into chemical energy during hydrolysis. ATP plays this role both in primitive and in higher organisms. This implies that the mechanism related to the participation of ATP appeared at a *very early stage of evolution*.

Many authors pointed out to the role of ATP as a source of energy. However from the standpoint of the suggested concept it is not less important that ATP hydrolysis is *conjugated* with reactions of biosynthesis. ATP hydrolysis is accompanied by the uptake of a water molecule, whereas the majority of reactions of synthesis proceed with the release of water:

$$\begin{array}{c} \text{ATP}+{\text{H}}_{2}\text{O}\to \text{ADP}+{\text{P}}_{\text{i}}\\ \text{M}+\text{N}\to {\text{H}}_{2}\text{O}+\text{MN} \end{array}$$

**Figure 2.**
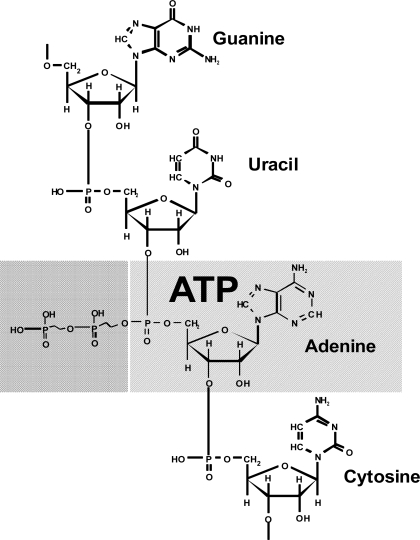
Adenosine is a structural unit of RNA.

For instance, formation of peptide bonds proceeds with the release of water, polymerization of nucleotides proceeds with the release of water, that is, the H_2_O molecule is a conjugated component. Conjugation of the ATP hydrolysis with the reactions of biosynthesis makes ATP an appropriate agent of ordering. It is very intriguing that adenosine phosphate (actually in the monophosphate form) is a structural unit of RNA (Figure 2).

The adenosine structure is part of the structure of many important biochemical compounds, for instance, DNA (in the desoxy form), NADP, FAD, coenzyme A, and others. This suggests that ATP originated *before* the formation of nucleic acids and, consequently, the genetic code. *i.e.*, ATP is a substance formed during the prebiological stage of evolution. Therefore I believe that appearance of ATP, rather than the “RNA - world”, as the extant paradigm suggests, is an initial point of emergence of life.

In spite of the fact that ATP is a rather complex molecule, both of its organic moieties: nucleic base adenine and sugar ribose have very simple precursors - HCN and HCHO respectively. Hydrogen cyanide and formaldehyde are widespread organic compounds in interstellar space, comets, and are readily synthesized in a reducing environment. Adenine was first synthesized by Oro [15] in electric spark experiments in an atmosphere of methane and ammonium. Sugars, including ribose, can be synthesized during formaldehyde condensation. This process is known as the formose reaction, which was first studied by Butlerov. ATP was synthesized by Ponnamperuma *et al.* [16] from a mixture of adenine, ribose, and phosphate, although with a very low yield of 0.05%.

In fact, the attractive simplicity of ATP synthesis is not easily realized in Nature. Coexisting HCN and HCHO may interact with each other, via the Strecker reaction [17], which produces important organic substances: amino and hydroxy acids, but blocks the pathway of adenine and ribose synthesis. The difficulty can be circumvented if one assumes separate synthesis of adenine and ribose. Under geologic conditions, this implies synthesis of adenine in a *reducing* atmosphere containing methane and molecular nitrogen, whereas ribose forms in an aquatic environment. It has been shown that borate minerals selectively stabilize ribose [18,19]. Ribose was also obtained in significant yield from glycolaldehyde phosphate [20,21]. It implies early phosphorylation of glycolaldehyde. Phosphorylation becomes efficient in the presence of ammonia [22]. Thus a strongly reducing atmosphere is favorable for ATP formation. Adenine structure contains no one oxygen atom. Therefore its synthesis in the reduced primitive atmosphere must have preceeded synthesis of any other nucleosides, i.e. GTP.

In addition, when ATP occurs in a water medium, its capacity of chemical conjugation with synthesis reactions becomes insignificant. Therefore, an anhydrous environment would be most favorable for ATP hydrolysis. The presence of water is, however, important in other respects, in particular, for the mobility of reactants. Therefore *alteration of the dry/wet* conditions, for instance, a periodically flooded bank, is a favorable environment for the ATP-based processes, as well as temperature cycling.

## 4. Functioning of the Ordering Unit

**Figure 3.**
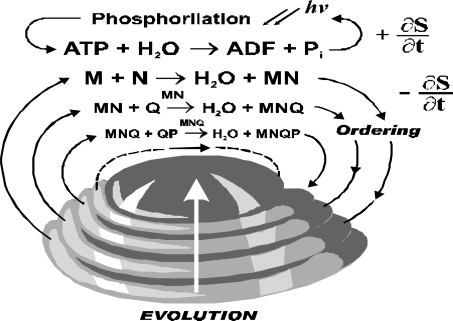
The model of ATP-related ordering.

ATP hydrolysis provides energy and it couples with a reaction producing negative entropy. It is cycled through photo-phosphorylation (Figure 3). Though the ATP hydrolysis is located in organism the model consider this reaction as a part of environment. The ordering reaction is cycled through reservoir of organic compounds, and even if it is external to a particular organism it is considered as part of the living system.

According to the definition formulated above ordering is restriction of both freedom of movement and freedom of interaction. A catalyst is an agent, which main property is restriction of interactions: only molecules of a certain chemical composition, structure, and chirality can interact. Selectivity is a property of a catalyst as well important as its property to accelerate a chemical reaction. Peptides are very efficient organic catalysts. In present-day life forms, they form protein enzymes that control almost all biochemical processes in living organisms. But even short amino acid chains manifest amazingly high catalytic activity. No natural substances perform ordering more efficiently than peptides.

However amino acid sequences are not capable of self-replication. Therefore, the ordering process solely based on peptide synthesis would have no evolutionary prospective. Unlike the peptides, nucleotide chains are able to self-replicate due to the complementary structure of *purines* and *pyrimidines.* Thus, two important properties necessary for evolution: the capability of ordering and capability of replication appeared to be separated between two classes of natural compounds. The evolving ordering has found a way to combine them. Peptides are copied indirectly by agency of nucleotides. This required translating amino acid sequences into the ‘language’ of nucleotide sequences. Each amino acid must find its reflection as a combination of nucleotides *(codon).* This correspondence is known under the name of *genetic code.*

Thus the genetic *code* is not an initial (mysterious?) information message, but just a bypass way, that Nature has found for reproducing the main carriers of ordering – peptides.

Nucleotide chains are also known to show some catalytic activity [23]. The discovery of the catalytic properties of RNA molecules fostered the “RNA world” hypothesis [24,25], which postulated the fundamental importance of RNA in a prebiological world. However, the catalytic activity of RNA is not comparable with that of peptides, and “RNA - world” hypothesis does not explain the logic of emergence of the genetic code.

## 5. Evolution

It has been shown above that ordering has an attractor and it can proceed in the linear field of irreversible processes. Unlike ordering, evolution has no attractor. One cannot point out any final stable state of evolution.

**Figure 4.**
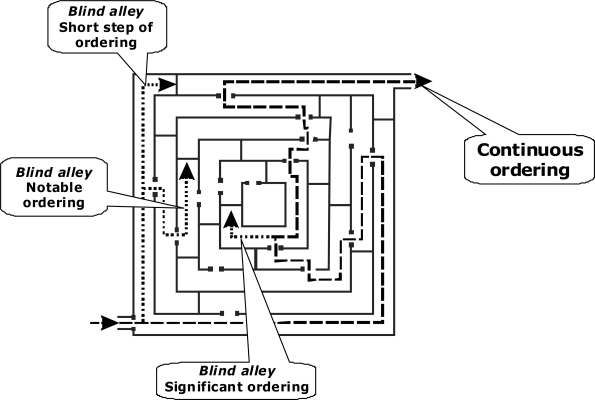
Straying in a labyrinth as an illustration of evolving ordering.

Production of ordering by restriction of freedom does not guarantee evolution of ordering of that type, which life demonstrates. Seeing this I argue that the biological evolution, which we observe, is due to limited range of possible ways by which ordering can be implemented. Indeed, the suggested concept of sustained ordering is applicable not only to the life phenomenon but to the inorganic world as well. But in inorganic world ordering cannot go too far. After one or two steps it meets a dead end (Figure 4). In contrast, life finds its way of developing over billions of years. This is caused by the unique features of carbon. The product with effective evolutionary advantage is not that which is more stable or produced in a larger yield, but that which provides a link to the subsequent ordering. This is like straying in a labyrinth: the correct step is not where one can comfortably stay but that which opens the way further. Of all the chemical elements only carbon possesses properties providing for the structures and functions of living matter - including biopolymer structures, enzymatic catalysis and the ability of molecular reproduction. This provides combinatorial ability incomparable to the other elements. On other hand the evolutionary capable combinations seem to be limited: Among organic carbon compounds only peptides can form universally structured (but also capable of presenting an endless variety) types of selective catalysis. Only nucleotides have the ability of self-reproduction — unique among organic compounds. Uniqueness of function is a property of many other biologically significant structures and carbon compounds. Therefore evolution of ordering looks to the certain degree to be invariant. Of course, branching exists. But the extant molecular structuring as well as entire phylogenetic tree is concluded in the narrow range of possible evolutionary capable variants compared to endless amount of conceivable combinations of ordering. For the same reason I argue that wherever in our Universe life might appear, the principles of its implementation must be similar. At the molecular level the life should have a similar structure in different worlds. Hypothetical life built according to different principles, out of different chemical elements and based on different chemical compounds rather than in its protein-nucleotide form is unlikely.

It is easy to see that concept of sustained ordering suggests principles of evolution different of the Darwinian selection. As example, Darwinism suggests evolution by small changes (mutations) which in every step are tested and fixed by natural selection. The efficient way of sustained ordering is a combination of already existent ordered structures. Combinatorial evolution can proceed not only through small changes, but also by *jumps*. It suggests the significant role of horizontal gene transfer. On other hand, combinations are formed from those sets, which are available at the moment. This suggests that some important combinations may be missing. Some forms and variants of ordering, which were in principle feasible, could be omitted. The sustained ordering tends to preserve obtained forms of ordering in succeeding generations. This leads to *evolutionary conservatism*. For example, the function of ATP hydrolysis, despite of the significant modification of its biochemical make-up, has been preserved from the very beginning to the present. The principle of competition in struggle for survival underlying Darwinism assumes that only the best fitted individuals survive. The sustained ordering does not require elimination or extrusion of the predecessor.

Natural selection plays its incident role. It controls a tendency toward disordering and degradation, when more stable components have selective advantage. Natural selection cannot lead to higher organized forms, but it is an important agent of adaptation, diversification and allocation of life.

In addition, the ATP-related model gives an idea of parameters favorable for the emergence of life. These are: highly reduced conditions, separate existence of atmosphere and water basins, alteration of dry/wet conditions and cycling of temperature. These are features of a differentiated planet rather than meteorites or other small celestial bodies. Recently it has been shown, unlike to the previous belief, that the reduced atmosphere is a common feature for the early stages of the terrestrial planets [26–29].

## 6. Conclusions

There exists an **attractor of ordering**, which appears in a steady state of conjugated reactions, involving entropy disproportionation and proceeding in the domain of linear interactions. This is a physical cause of a natural ***sustained ordering*** process, which is a driving force of emergence and evolution of life.

***Adenosine triphosphate*** **(ATP)** played the key role in pre-biological times for the realization of this mechanism.

The observed biological evolution is due to limited range of possible ways by which ordering can be implemented. The life is product of the unique features of **carbon chemistry**.

The model suggests certain **testable principles** of biological evolution.
